# Cytotoxicity of four *Aframomum* species (*A. arundinaceum, A. alboviolaceum, A. kayserianum* and *A. polyanthum*) towards multi-factorial drug resistant cancer cell lines

**DOI:** 10.1186/1472-6882-14-340

**Published:** 2014-09-19

**Authors:** Victor Kuete, Patrick Y Ango, Samuel O Yeboah, Armelle T Mbaveng, Renameditswe Mapitse, Gilbert DWF Kapche, Bonaventure T Ngadjui, Thomas Efferth

**Affiliations:** Department of Pharmaceutical Biology, Institute of Pharmacy and Biochemistry, University of Mainz, 55128 Mainz, Germany; Department of Chemistry, Faculty of Science, University of Botswana, Francistown, Botswana; Departments of Biochemistry, Faculty of Science, University of Dschang, Dschang, Cameroon; Departments of Organic Chemistry, Faculty of Science, University of Yaoundé I, Yaoundé, Cameroon; Department of Chemistry, Higher Teachers’ Training College, University of Yaoundé I, Yaoundé, Cameroon

**Keywords:** *Aframomum*, Cameroon, Cancer, Cytotoxicity, Multidrug resistant, Zingiberaceae

## Abstract

**Background:**

The search for natural products as potential cytotoxic agents has yielded promising candidates. However multidrug resistance (MDR) is still a major hurdle for patients receiving chemotherapy. In the present study, we evaluated the cytotoxicity of the methanol extracts of four dietary *Aframomum* plant species (*A. arundinaceum, A. alboviolaceum, A. kayserianum* and *A. polyanthum*) against nine sensitive and MDR cancer cell lines. We have also identified the bioactive constituents of *A. arundinaceum*.

**Methods:**

The cytotoxicity of the methanol extracts of the above plants was determined using a resazurin reduction assay. Chromatographic techniques were used to isolate the constituents of *A. arundinaceum.*

**Results:**

A preliminary experiment on leukemia CCRF-CEM cells at 40 μg/mL showed that the extracts from *A. kayserianum* and *A. alboviolaceum* as well as the isolated compounds namely compounds aframodial (**1**), 8(17),12-labdadien-15,16-dial (**2**), galanolactone (**3**), 1-*p*-menthene-3,6-diol (**6**) and 1,4-dimethoxybenzene (**7**) were less active, inducing more than 50% growth of this cell line contrary to *A. polyanthum* and *A. arundinaceum* extracts, galanals A (**4**) and B (**5**), naringenin (**8**) and kaempferol-3,7,4’-trimethylether (**9**). The IC_50_ values below or around 30 μg/mL were recorded with *A. arundinaceum* extract against eight of the nine tested cancer cell lines. This extract as well as compound **8** displayed IC_50_ values below 40 μg/mL towards the nine tested cancer cell lines whilst *A. polyanthum* extract, compounds **4, 5** and **9** showed selective activities. Collateral sensitivity (hypersensitivity) was observed with *A. arundinaceum* extract towards leukemia CEM/ADR5000 cells and glioblastoma U87MG.*ΔEGFR* compared to their respective sensitive counterparts CEM/CEM and U87MG.

**Conclusion:**

The results of this study provide evidence of the cytotoxicity selected *Aframomum* species as well as a baseline information for the potential use of *Aframomum arundinaceum* in the fight against drug sensitive and otherwise drug-resistant cancers.

## Background

Chemotherapy remains the major treatment of cancers but often fails due to cells multidrug resistance (MDR) [1, 2]. MDR is displayed by many cancer cells to withstand increasingly higher doses of antineoplastic compounds [3]. Investigation for naturally occurring molecules as potential cytotoxic drugs has yielded promising candidates [3–7]. However, MDR is still considered a major hurdle for patients receiving chemotherapy [8, 9]. Various Cameroonian dietary plants including those from the family Zinziberaceae are used in traditional medicine to manage various ailments [5, 10–13]. The genus *Aframomum*, belonging to the Zingiberaceae family have 40 species and is most common in tropical and subtropical regions [14]. Twenty species are found in Cameroon, where they are widely used as spices and in traditional medicine [14]. The Seeds of *Aframomum arundinaceum* K. Schum are used as laxative and as anti-helmintic. The fresh juice of the rhizomes is used against body odor. The rhizomes are used against toothache and the crushed seeds against fungal infections [10]. The decoction of the leaves *Aframomum melegueta* K. Schum together with the leaves of *Momordica charantia* and *Sorghum arundinaceum* cereal in local dry gin (alcohol) is recommended to be taken one dose daily against cholera [15]. Several *Aframomum* species such as *Aframomum angustifolium, A. danielli, A. sanguineum,* and *A. sulcatum* are also traditionally used to treat fevers in Africa [16], and recently, the antiplasmodial activity of some labdanes from *A. sceptrum* and *A. latifolium* was demonstrated [17]. The antibacterial activities of *Aframomum kayserianum* [12] and *Aframomum polyanthum* [13] were also reported on Gram-negative multidrug-resistant phenotypes. The cytotoxicity of other *Afromomum* species such as *A. citratum* and *A. melegueta* towards leukemia CCRF-CEM and ADR5000 cell lines was also reported [5]. The present study was designed to investigate the cytotoxicity of four dietary *Aframomum* species commonly used as spices in Cameroon, *Aframomum alboviolaceum* (Ridl.) K. Schum, *A. arundinaceum* (Oliver & Hanbury) K. Schum*, Aframomum kayserianum* K. Schum and *Aframomum polyanthum* K. Schum towards sensitive and multi-factorial drug resistant cancer cell lines. The study was extended to the identification of the bioactive constituents of *A. arundinaceum*.

## Methods

### Plant material and extraction

The tested *Aframomum* species, *A. alboviolaceum, A. kayserianum* and *A. polyanthum* were purchased from Bafoussam local market (West region of Cameroon) in January 2012. *Aframomum arundinaceum* was collected in Yaoundé (Centre region) in March 2012. The plants were further identified at the National Herbarium (Yaoundé, Cameroon) where voucher specimens were deposited under the reference numbers 11704/SFR/CAM (*A. arundinaceum*), 34888/HNC (*A. alboviolaceum*), 18884/SRFC (*A. kayserianum*) and 3981/SRFK (*A. polyanthum*). The air dried fruits of *A. kayserianum, A. polyanthum* (100 g) and *A. arundinaceum* (3000 g) as well as the roots of *A. alboviolaceum* (100 g) were powdered and macerated with methanol for 48 h at room temperature. The methanol extract was concentrated in *vacuo* to give 18.7 g, 21.2 g, 25.3 and 100 g of the crude extracts of *A. kayserianum, A. polyanthum*, *A. alboviolaceum* and *A. arundinaceum* respectively. The extracts were then conserved at 4°C until further use.

### Isolation of compounds from Aframomum arundinaceum

Crude extract of *A. arundinaceum* (100 g) was successively extracted with petroleum ether, chloroform and methanol at room temperature. The petroleum ether fraction (25 g) was column chromatographed on 100 g of silica gel (Merck, 0.040-0.063 mm) using hexane and hexane-choloroform mixture with increasing polarity. Fractions of 300 mL were collected, concentrated, and pooled on the basis of their thin layer chromatography (TLC) profile. The obtained fractions (frs) directly afforded a yellow oil (**1;** frs 4 to 9; 30 mg) and amorphous powders **2** ( frs 13 to 16; 25 mg), **3** (frs 20 to 27; 40 mg), **4** (frs 30 to 33) and **5** (frs 36 to 38; 10 mg). The chloroform extract (20 g) was also column chromatographed on 250 g of silica gel (Merck, 0.040-0.063 mm) using hexane (Hex) and mixture of hexane-chloroform (Hex-CHCl_3_). Fractions of 400 mL were collected, concentrated and pooled after TLC analysis to give five sub-fractions (sub-frs A-E).

Sub-fraction B (Hex-CHCl_3_; 10 to 25; 6 g) was subjected to column chromatography (CC) to afford a white crystal (**6;** 20 mg). Sub-fraction C (8.0 g) obtained with Hexane-CHCl_3_ 4:6 was subjected to CC (silica gel 60, 50 g) and eluted with Hex-CHCl_3_ mixtures of increasing polarity to give 6 new sub-fractions (C_1_-C_6_). Sub-fraction C_4_ obtained with Hex-CHCl_3_ 6:4. afforded a yellow oil (**7;** 10 mg). Sub-fraction C_5_ (Hex-CHCl_3_ 4:6) and C_6_ (Hex-CHCl_3_ 8:2) were repeatedly filtered through Sephadex LH-20 (CHCl_3_-MeOH 7:3) to give yellow powders, **8** (sub-frs 3 to 6; 10.0 mg) and **9** (sub-frs 15 to 19; 15 mg).

### General procedure

**Figure 1:**
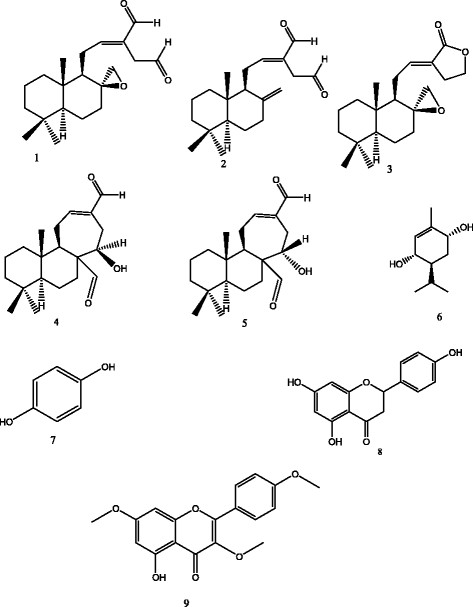
**Chemical structures of compounds isolated from the fruit of** ***Aframomum arundinanceum*** **K Schum. 1**: aframodial; **2**: 8(17),12-labdadien-15,16-dial; **3**: galanolactone; **4**: galanal A; **5**: galanal B; **6**: 1-*p*-menthene-3,6-diol; **7**: 1,4-dihydroxybenzene; **8**: naringenin; **9**: kaempferol-3,7,4’-trimethylether.

Aluminum sheet pre-coated with silica gel 60 *F*254 nm (Merck) was used for thin layer chromatography; the spots were visualized using both ultraviolet light (254 and 366 nm) and 50% H_2_SO_4_ spray reagent. NMR spectra were recorded on a Bruker Avance 300 (Billerica, MA, USA) at 300 MHz (^1^H) and 75 MHz (^13^C), with the residual solvent peaks as internal references. Mass spectra were recorded with API QSTAR pulsar mass (Milford, MA, USA). Melting points (m.p) were recorded using a Stuart Scientific (Redhill, Surrey, UK) melting point apparatus (SMP1) and are uncorrected. The chemical structures of the compounds were confirmed by comparing with reference data from available literature (Figure 1).

#### Chemicals

Doxorubicin 98.0% were provided by the University Pharmacy of the Johannes Gutenberg University (Mainz, Germany) and dissolved in PBS (Invitrogen, Eggenstein, Germany) at a concentration of 10 mM. Geneticin >98% (72.18 mM; Sigma-Aldrich, Munich, Germany).

#### Cell cultures

The cell lines used the present work, their origins and their treatments were previously reported [18, 19]. They include drug-sensitive CCRF-CEM and multidrug-resistant P-glycoprotein over-expressing CEM/ADR5000 leukemia cells [20–22], the MDA-MB-231-pcDNA3 breast cancer cells and its resistant subline MDA-MB-231-*BCRP* clone 23) [23], the HCT116 (*p53*^*+/+*^) colon cancer cells and its knockout clones HCT116 (*p53*^*-/-*^), the U87MG glioblastoma cells and its resistant subline U87MG.Δ*EGFR,* HepG2 hepatocarcinoma cells and AML12 normal hepatocytes [6, 19, 24]. The CCRF-CEM and CEM/ADR5000 leukemia cells were maintained in RPMI 1640 medium (Invitrogen) supplemented with 10% fetal calf serum in a humidified 5% CO_2_ atmosphere at 37°C. Sensitive and resistant cells were kindly provided by Dr. Axel Sauerbrey (Department of Pediatrics, University of Jena, Jena, Germany). The generation of the resistant subline was previously described [6, 19, 24]. Breast cancer cells, transduced with control vector (MDA-MB-231-pcDNA3) or with cDNA for the breast cancer resistance protein *BCRP* (MDA-MB-231-*BCRP* clone 23), were maintained under standard conditions as described above for CCRF-CEM cells. Human wild-type HCT116 (*p53*^*+/+*^) colon cancer cells as well as knockout clones HCT116 (*p53*^*-/-*^) derived by homologous recombination were a generous gift from Dr. B. Vogelstein and H. Hermeking (Howard Hughes Medical Institute, Baltimore, MD). Human glioblastoma multiforme U87MG cells (non-transduced) and U87MG cell line transduced with an expression vector harboring an epidermal growth factor receptor (*EGFR*) gene with a genomic deletion of exons 2 through 7 (U87MG.Δ*EGFR*) were kindly provided by Dr. W. K. Cavenee (Ludwig Institute for Cancer Research, San Diego, CA). MDA-MB-231-*BCRP,* U87MG.Δ*EGFR* and HCT116 *(p53*^*-/-*^*)* were maintained in DMEM medium containing 10% FBS (Invitrogen) and 1% penicillin (100 U/mL)-streptomycin (100 μg/mL) (Invitrogen) and were continuously treated with 800 ng/mL and 400 μg/mL geneticin, respectively. Human HepG2 hepatocellular carcinoma cells and normal AML12 heptocytes were obtained from the American Type Culture Collection (ATCC, USA). The above medium without geneticin was used to maintain MDA-MB-231, U87MG, HCT116 (*p53*^*+/+*^), HepG2 and AML12 cell lines. The cells were passaged twice weekly. All experiments were performed with cells in the logarithmic growth phase.

#### Resazurin reduction assay

The cytotoxicity of the studied samples was performed by resazurin reduction assay as we previously described [6, 18, 19, 24–26]. Briefly, adherent cells at 1x10^4^ cells were allowed to attach overnight and then treated with different studied samples. Samples were preliminary tested at 40 μg/mL (extract and isolated compounds) and doxorubicin (20 μg/mL) against the sensitive leukemia CCRF-CEM cell line and those inducing less than 50% growth proliferation were further tested for IC_50_ determinations towards all the studied cell lines. For suspension cells, aliquots of 2 × 10^4^ cells per well were seeded in 96-well-plates in a final volume of 200 μL. Extracts and compounds were prior diluted in DMSO and tested in a final concentration below 0.1% (A final concentration of 0.1% DMSO was used as negative control and did not show any effect on cell growth). The tested concentrations ranges were 0.16 μg/mL to 40 μg/mL for crude extracts and isolated compounds and 0.08 μg/mL to 20 μg/mL for doxorubicin. After 72 h incubation and a resazurin (Sigma-Aldrich, Schnelldorf, Germany) staining, fluorescence was measured on an Infinite M2000 Pro™ plate reader (Tecan, Crailsheim, Germany) using an excitation wavelength of 544 nm and an emission wavelength of 590 nm. Each assay was done at least two times, with six replicates each. IC_50_ values represent the sample’s concentrations required to inhibit 50% of cell proliferation and were calculated from a calibration curve by linear regression using Microsoft Excel [5, 6].

## Results and discussion

The structures of the compounds isolated from *Aframomum arundinaceum* were established using spectroscopic analysis, especially, NMR spectra in conjunction with 2D experiments, COSY, HMQC, HMBC, and direct comparison with published information and with authentic specimens obtained in our research group for some cases. The compounds isolated from the fruits of *A. arundinaceum* (Figure 1) were identified as Aframodial C_20_H_30_O_3_ (**1**; m/z 318.2) [27], 8(17),12-labdadien-15,16-dial C_20_H_30_O_2_ (**2**; m/z 302.2) [17], galanolactone C_20_H_30_O_3_ (**3**; m/z 318.2) [27], galanal A C_20_H_30_O_3_ (**4**; 15 mg, m/z 318.2) [28], and galanal B C_20_H_30_O_3_ (**5**; m/z 318.2) [29], 1-*p*-menthene-3,6-diol C_10_H_18_O_2_ (**6**; m/z 170.1; m.p:165-167°C) [30], 1,4-dihydroxybenzene C_6_H_6_O_2_ (**7**; m/z 110.0) [31], naringenin C_15_H_12_O_5_ (**8**; m/z 272.0; 245-248°C) [32] and kaempferol-3,7,4’-trimethylether C_18_H_16_O_6_ (**9**; m/z 328.0; 157-158°C) [33]. The cytotoxicity of compounds **1-9** as well as the crude extracts was determined towards drug sensitive and resistant cancer cell lines.

**Figure 2:**
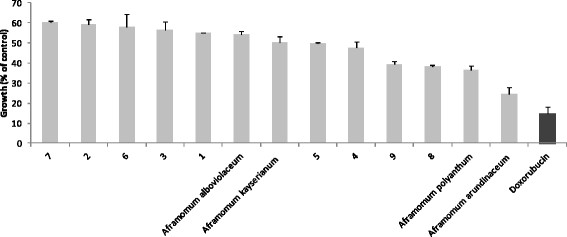
**Growth percentage (%) of leukemia CCRF-CEM cancer cell line treated with plant extracts and isolated compounds at 40 μg/mL and doxorubicin (20 μg/mL). 1**: aframodial; **2**: 8(17),12-labdadien-15,16-dial; **3**: galanolactone; **4**: galanal A; **5**: galanal B; **6**: 1-*p*-menthene-3,6-diol; **7**: 1,4-dihydroxybenzene; **8**: naringenin; **9**: kaempferol-3,7,4’-trimethylether.

In a preliminary investigation of the four studied *Aframomum* species and compounds isolated from *A. arundinaceum*, we tested a single concentration of 40 μg/mL for each sample and 20 μg/mL for doxorubicin against the sensitive CCRF-CEM leukemia cell line (Figure 2). The extracts from *A. kayserianum* and *A. alboviolaceum* were less active and induced respectively 50.33% and 54.36% growth proliferation of CCRF-CEM cells. Compounds **1, 2, 3, 6** and **7** also induced more than 50% growth of this cell line. The extracts from *A. polyanthum* (36.28%) and *A. arundinaceum* (24.68%) as well as compounds **4** (47.78%), **5** (49.81%), **8** (38.49%) and **9** (39.58%) displayed less than 50% growth proliferation of CCRF-CEM cells. The IC_50_ values of the above samples were further determined on nine cancer cell lines, including both sensitive and MDR phenotypes (Table 1). *Aframomum. arundinaceum* extract as well as compound **8** and doxorubucin induced less than 50% proliferation of all tested cancer cell lines, with IC_50_ values below 40 μg/mL. *A. polyanthum* extract, compounds **9, 4** and **5** showed selective activities, the IC_50_ values <40 μg/mL being obtained on 5/9, 4/9, 2/9 and 1/9 tested cell lines respectively (Table 1). According to the National Cancer Institute (USA), 30 μg/mL is the upper IC_50_ limit considered promising for purification of a crude extract [34]. We therefore, tested a slightly higher concentration of 40 μg/mL in our preliminary assay. Also, the IC_50_ threshold value of 4 μg/ml or 10 μM [35, 36] after 48 and 72 h incubations has been set to identify good cytotoxic compounds. Considering these thresholds, the IC_50_ values below or around 30 μg/mL were recorded with *A. arundinaceum* extract against eight of the nine tested cancer cell lines (Table 1) explaining why it was considered further for purification. Nonetheless, the extract from *A. polyanthum* also showed activities with IC_50_ values <30 μg/mL on four of the nine tested cancer cell lines. Though Compound **8** was active on all the tested cancer cell lines, no IC_50_ below 4 μg/ml was recorded, the lowest values being 7.86 μg/mL against CEM/ADR5000 cells. Interestingly, none of the selected extracts and compounds was more toxic towards AML12 normal hepatocytes (IC_50_ > 40 Mg/mL) than cancer cell lines, suggesting their good selectivity. Importantly, collateral sensitivity (hypersensitivity) was also observed with *A. arundinaceum* extract towards CEM/ADR5000 cells (degree of resistance of 0.76) and U87MG.*ΔEGFR* (degree of resistance of 0.95) compared to their respective sensitive counterparts CEM/CEM and U87MG. This extract was also more active against hepatocarcinoma HepG2 as compared to AML12 normal hepatocytes, confirming its selectivity to cancer cells (Table 1). Despite the fact that compound **8** showed moderate activities, it also displayed better collateral sensitivity of MDR cell lines compared to doxorubicin. The use of natural products to fight multidrug resistance is an attractive strategy in chemotherapy [37–39]. P-gp-expressing CEM/ADR5000 as well as p53 knock out HCT116 (*p53*^*-/-*^) and *BCRP-*expressing U87MG.Δ*EGFR* cells were less cross-resistant towards the best samples namely *A. arundinaceum* and compound **8** than towards the positive drug, doxorubicin, highlighting their possible therapeutic potential in the fight against multidrug resistance. This report also highlights the importance of the plants of the genus *Aframomum* as potential source of cytotoxic compounds. The results obtained collaborate with previous investigations. In effect, *Aframomum melegueta* previously inhibited the proliferation of the leukemia ADR5000 cell lines with a reported IC_50_ value of 7.80 μg/mL [5]. Also, naringenin (**8**) has shown cytotoxicity in various human cancer cell lines and induced apoptosis *via* a transient induction of caspase-3/CPP32 activity, in the human promyeloleukemia cell line HL-60 [40–42]. The moderate cytotoxicity of galanals A (**4**; IC_50_ of 18 μM or 5.62 μg/mL) and B (**5**; IC_50_ of 32 μM or 12.21 μg/mL) towards human T lymphoma Jurkat cells was also reported [29].

**Table 1:** **Cytotoxicity of the studied** ***Aframomum*** **extracts, compounds and doxorubicin towards sensitive and drug-resistant cancer cell lines and normal cells as determined by the resazurin assay**

Cell lines	Studied samples, IC _50_values (μg/mL) ^a^and degree of resistance (in braket)						
	***Aframomum***species		Compounds from ***A. arundinaceum***				Doxorubucin
	***A. polyanthum***	***A. arundinaceum***	4	5	8	9	
CCRF-CEM	20.37 ± 3.10	18.08 ± 0.98	17.32 ± 1.96	19.81 ± 2.01	12.20 ± 1.87	18.38 ± 2.04	0.11 ± 0.01
CEM/ADR5000	28.16 ± 1.24 (1.38)	13.73 ± 1.02 (0.76)	- (>2.31)	- (>2.02)	7.86 ± 0.74 (0.64)	18.22 ± 1.18 (0.99)	195.12 ± 14.30 (1772)
MDA-MB-231	33.79 ± 2.38	29.98 ± 1.86	-	-	9.51 ± 1.03	-	1.10 ± 0.01
MDA-MB-231-*BCRP*	30.24 ± 2.18 (0.89)	30.66 ± 3.17 (1.02)	27.99 ± 2.39 (<0.70)	-	18.12 ± 2.01 (1.91)	33.14 ± 2.64 (<0.83)	7.83 ± 0.01 (7.11)
HCT116 *p53* ^*+/+*^	-	23.06 ± 2.21	-	-	13.65 ± 1.11	-	1.43 ± 0.02
HCT116 *p53* ^*-/-*^	-	27.38 ± 1.92 (1.19)	-	-	13.86 ± 0.94 (1.02)	36.74 ± 2.31 (<0.82)	4.06 ± 0.04 (2.84)
U87MG	-	36.70 ± 2.12	-	-	29.81 ± 1.88	-	1.06 ± 0.03
U87MG.*ΔEGFR*	20.59 ± 1.87 (<0.51)	24.42 ± 1.95 (0.67)	-	-	18.02 ± 1.34 (0.60)	-	6.11 ± 0.04 (5.76)
HepG2	-	23.15 ± 1.97 (<0.58)	-	-	23.46 ± 1.95 (<0.59)	-	1.41 ± 0.12 (<0.04)
AML12	-	-	-	-	-	-	-

^a^The degree of resistance was determined as the ratio of IC_50_ value in the resistant divided by the IC_50_ in the sensitive cell line; AML12 was used as the corresponding resistant counterpart for HepG2. **4**: galanal A; **5**: galanal B; **8**: naringenin; **9**: kaempferol-3,7,4’-trimethylether; (-): >40 μg/mL.

## Conclusions

Finally, this work provides further evidence of the cytotoxic potential of *Aframomum* species and highlights the good activity of *Aframomum arundinaceum* on sensitive and drug-resistant cancer cell lines. Bioactive constituents of this plant include galanals A and B, naringenin and kaempferol-3,7,4’-trimethylether. *Aframomum arundinaceum* could be explored in more detail in the future to develop novel anticancer drugs against sensitive and resistant phenotypes.
